# Transthyretin Cardiac Amyloidosis With Reduced Ejection Fraction in a 47-Year-Old Man: An Unusual Presentation

**DOI:** 10.7759/cureus.101833

**Published:** 2026-01-19

**Authors:** Mehdi Moujahid, Idriss Allalat, Mouad Lamtai, Nadia Fellat

**Affiliations:** 1 Cardiology, Centre Hospitalo-Universitaire Ibn Sina, Rabat, MAR

**Keywords:** cardiac amyloidosis, non-invasive diagnosis, reduced ejection fraction, restrictive cardiomyopathy, transthyretin amyloidosis

## Abstract

Cardiac amyloidosis is an infiltrative cardiomyopathy caused by extracellular deposition of misfolded proteins, leading to restrictive physiology and, in advanced stages, systolic dysfunction. Although transthyretin amyloidosis most commonly affects older adults, earlier presentations should prompt consideration of hereditary disease and genetic evaluation. We report a 47-year-old man admitted with advanced heart failure manifested by anasarca and progressive dyspnea. Electrocardiography showed low-voltage QRS complexes discordant with the degree of wall thickening. Transthoracic echocardiography revealed concentric left ventricular thickening with markedly reduced left ventricular ejection fraction (35%), and speckle-tracking demonstrated a reduced global longitudinal strain with relative apical sparing. Technetium-labeled bone scintigraphy demonstrated Perugini grade 2 myocardial uptake in the absence of monoclonal gammopathy, strongly supporting transthyretin cardiac amyloidosis (ATTR-CM). Tafamidis was initiated, and genetic testing was requested; however, results were not available at the time of manuscript submission. This case highlights that ATTR-CM should be considered in patients with unexplained heart failure and increased ventricular wall thickness even at a relatively young age, and that prompt diagnosis enables timely initiation of targeted therapy and supports genetic counseling and family screening when hereditary disease is suspected.

## Introduction

Cardiac amyloidosis is an uncommon but frequently overlooked etiology of heart failure. It results from extracellular deposition of misfolded proteins in the myocardium, leading to progressive stiffening of the ventricular walls, impaired filling, and, in later stages, systolic dysfunction. The two major entities responsible for cardiac involvement are immunoglobulin light-chain (AL) amyloidosis and transthyretin (TTR) amyloidosis [1].

TTR amyloidosis develops when transthyretin, a hepatic transport protein for thyroxine and retinol-binding protein, destabilizes and misfolds. Two forms are recognized: hereditary ATTR (hATTR), caused by pathogenic variants in the TTR gene, and wild-type ATTR (wtATTR), which predominantly affects older men and was historically described as senile systemic amyloidosis [2,3]. Although wtATTR is typically identified in the elderly, hATTR may present earlier, often before 60 years of age, and its phenotype varies with the underlying mutation, ranging from predominantly cardiac to neurologic or mixed disease [4]. Accordingly, transthyretin cardiac amyloidosis (ATTR-CM) presenting before 60 years of age is uncommon and should raise suspicion for hereditary disease, making genetic evaluation particularly important in younger patients [3,4].

From a clinical standpoint, cardiac amyloidosis most often manifests as heart failure with preserved ejection fraction (HFpEF) driven by restrictive physiology. Nevertheless, reduced left ventricular ejection fraction (LVEF) can occur, particularly in advanced ATTR or in association with more aggressive disease patterns [5]. The diagnostic pathway has evolved markedly with the broader use of technetium-labeled bone scintigraphy. In the appropriate clinical context and after exclusion of monoclonal gammopathy, myocardial tracer uptake can support the diagnosis of ATTR-CM and may allow clinicians to avoid endomyocardial biopsy [6]. Early recognition is essential because disease-modifying therapy, most notably tafamidis, can improve outcomes by stabilizing the transthyretin tetramer and slowing disease progression [7].

We present the case of a 47-year-old man with advanced heart failure and markedly reduced LVEF who was diagnosed with ATTR-CM. This report highlights the need to consider cardiac amyloidosis even in younger patients with otherwise unexplained cardiomyopathy and emphasizes the role of non-invasive imaging and genetic evaluation when hereditary ATTR is suspected.

## Case presentation

A 47-year-old man with no prior medical history and no known cardiovascular risk factors presented to the emergency department with progressive dyspnea and generalized edema evolving over several weeks. On admission, he had marked anasarca with bilateral lower-extremity edema up to the knees, abdominal distension, and exertional shortness of breath. Physical examination revealed jugular venous distension, bilateral mid-zone crackles, ascites, and significant pitting edema. He was in the New York Heart Association (NYHA) functional class IV [8]. Vital signs showed a blood pressure of 105/65 mmHg (reference value: <120/80 mmHg) and an oxygen saturation of 93% on room air (reference value: ≥95%).

**Figure 1:**
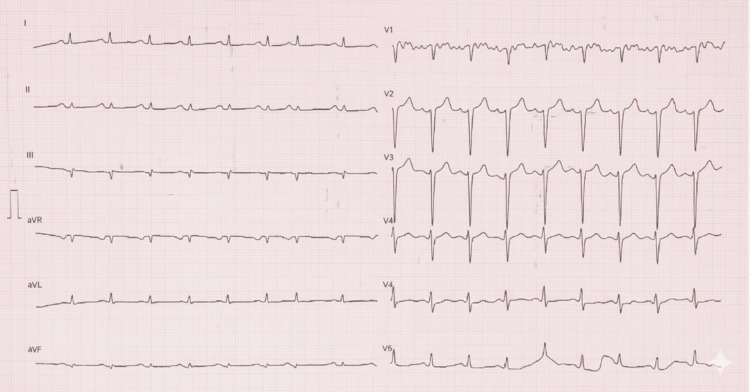
Twelve-lead electrocardiogram Demonstrates sinus tachycardia with low-voltage QRS complexes in the limb leads and poor R-wave progression in the precordial leads, findings suggestive of an infiltrative cardiomyopathy such as cardiac amyloidosis.

Baseline electrocardiography showed sinus tachycardia with low-voltage QRS complexes in the limb leads and poor R-wave progression in the precordial leads, discordant with the degree of left ventricular wall thickening suspected clinically (Figure 1).

Given the presence of right atrial thrombus, continuous in-hospital rhythm monitoring was performed; however, ambulatory Holter monitoring was not performed.

**Figure 2:**
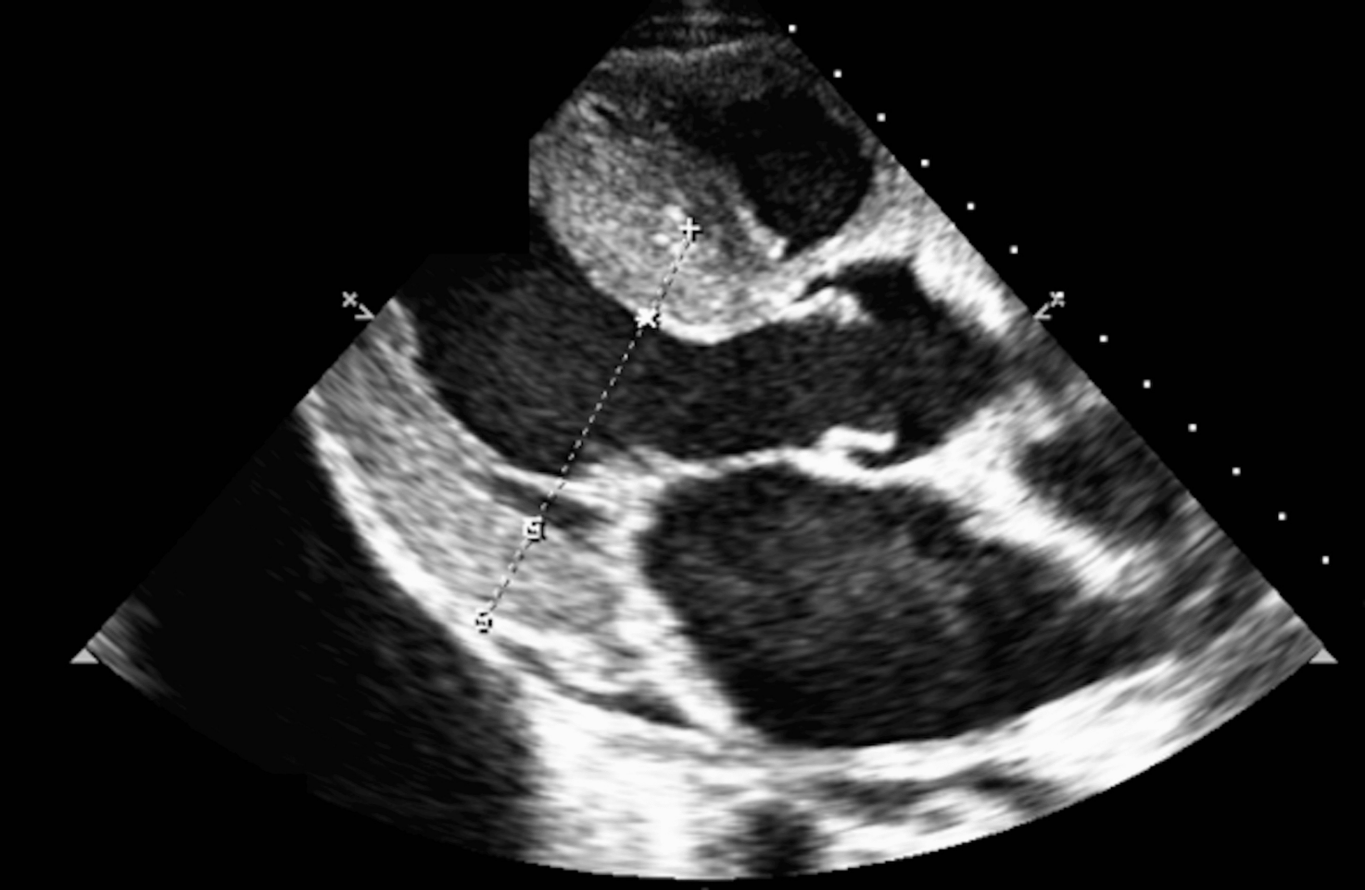
Parasternal long-axis echocardiographic view at the level of the papillary muscles Demonstrates marked left ventricular wall thickening (interventricular septum 1.61 cm; posterior wall 1.67 cm), consistent with an infiltrative cardiomyopathy. The left ventricular cavity appears small.

Transthoracic echocardiography demonstrated a thickened left ventricle (interventricular septum 16 mm; posterior wall 17 mm) with diffuse hypokinesia and a reduced LVEF of 35% (Figure 2).

**Figure 3:**
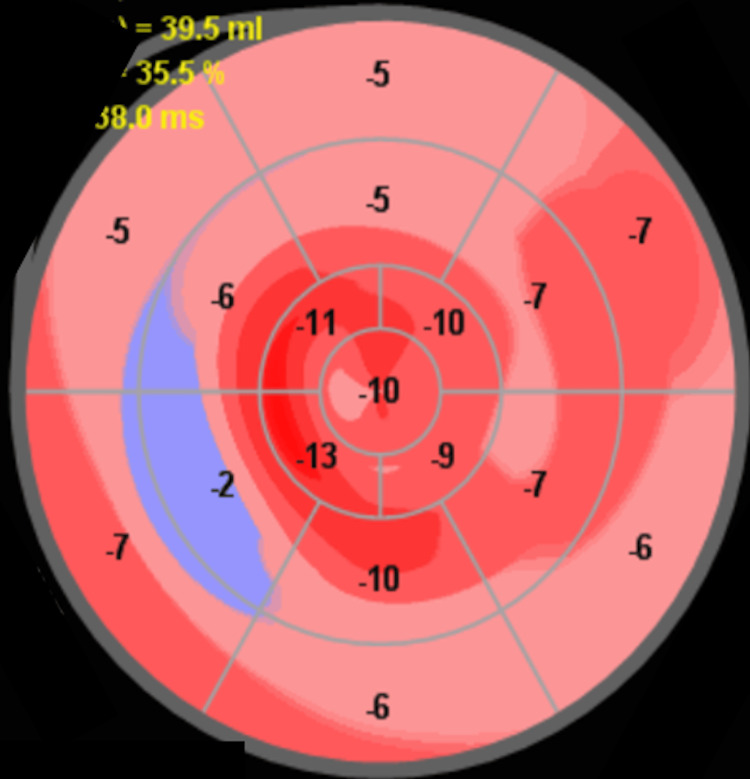
Bull's-eye plot of the global longitudinal strain (GLS) by speckle-tracking echocardiography The polar map demonstrates severely reduced global longitudinal strain (GLS: –7%) with relative preservation of apical segments ("apical sparing" pattern), which is characteristic of cardiac amyloidosis.

The interventricular septum appeared flattened. The right ventricle was mildly hypertrophied (7 mm) with borderline longitudinal systolic function (tricuspid annular S′ 9.8 cm/s). Both atria were severely enlarged, and spontaneous echocardiographic contrast (“smoke”) was observed in the left atrium. Tricuspid regurgitation peak velocity was 3.10 m/s, corresponding to an estimated systolic pulmonary artery pressure of approximately 45 mmHg. The inferior vena cava was dilated (28 mm) with reduced inspiratory collapse, and a small pericardial effusion was present, supporting a restrictive cardiomyopathy phenotype. Speckle-tracking echocardiography showed severely reduced global longitudinal strain with relative apical sparing, a pattern characteristic of cardiac amyloidosis (Figure 3).

**Figure 4:**
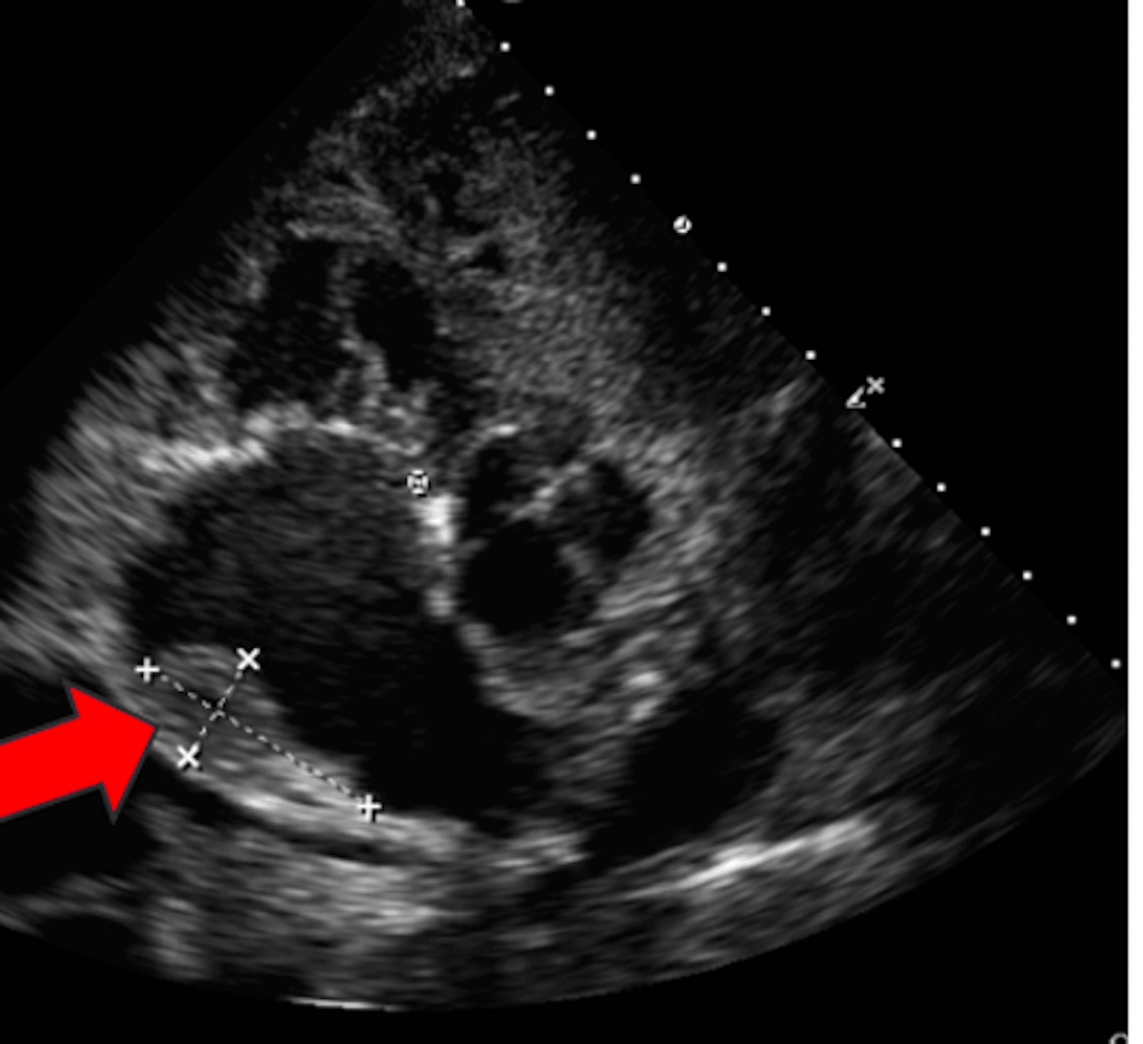
Parasternal short axis view showing a right atrial thrombus A non-mobile echogenic mass is visualized in the right atrium, consistent with thrombus formation. This finding may reflect severe atrial stasis secondary to restrictive cardiomyopathy in the context of cardiac amyloidosis.

In addition, a non-mobile echogenic mass within the right atrium, consistent with an intracavitary thrombus, was identified (Figure 4).

Laboratory testing revealed elevated N-terminal pro-B-type natriuretic peptide (NT-proBNP of 4,300 pg/mL; reference range: <450 pg/mL for patients <55 years), with mildly increased cardiac troponin levels (above the upper reference limit of the local laboratory), while renal and hepatic functions remained preserved. Serum and urine protein electrophoresis with immunofixation were negative, and the serum free light-chain ratio was within normal limits, arguing against AL amyloidosis.

The patient was treated with intravenous diuretics for decongestion and therapeutic anticoagulation with rivaroxaban for right atrial thrombosis. This decision was further supported by the increased thromboembolic risk associated with cardiac amyloidosis due to atrial enlargement and mechanical dysfunction.

**Figure 5:**
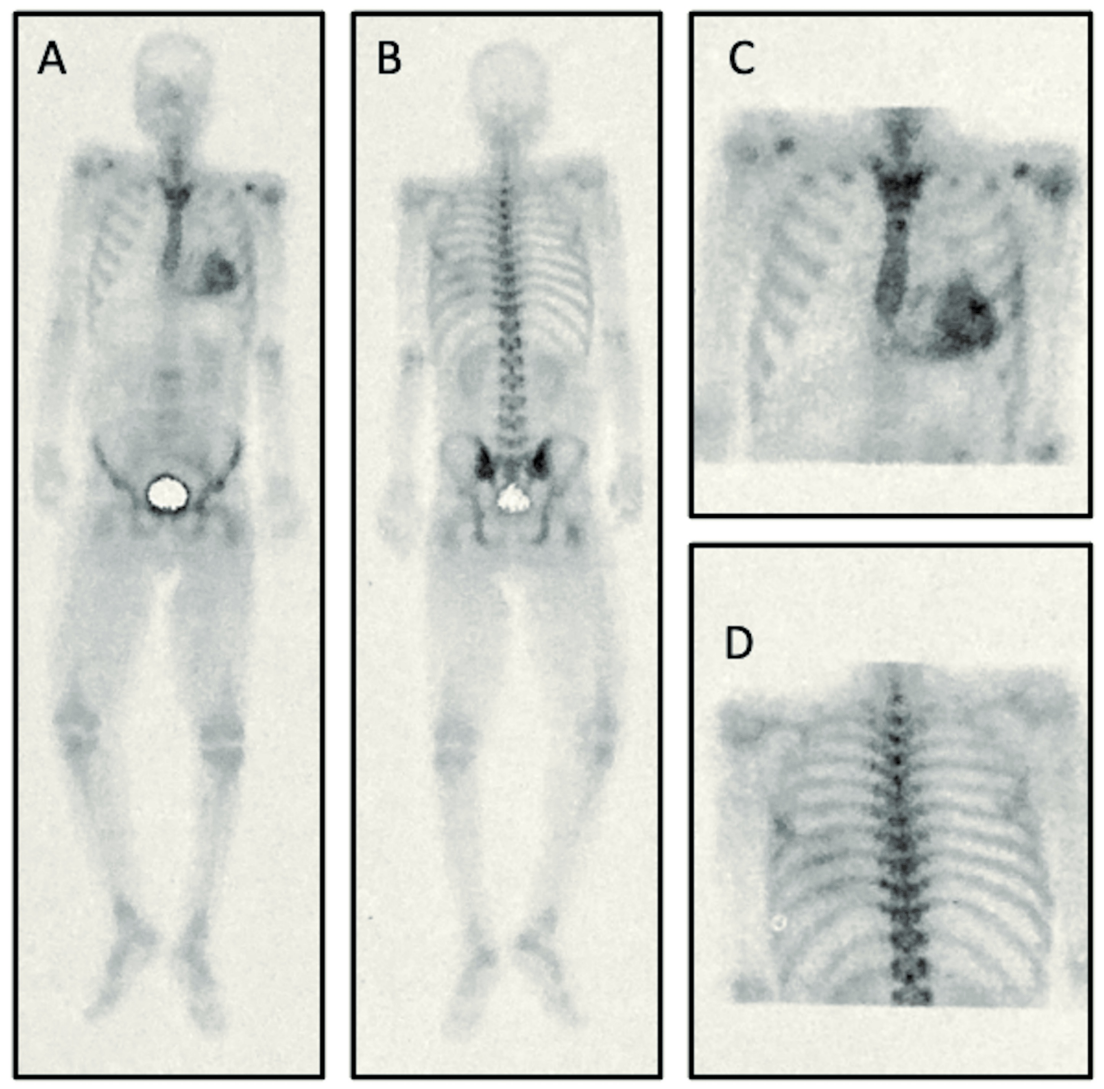
99mTc-DPD scintigraphy showing myocardial uptake suggestive of transthyretin cardiac amyloidosis (A) Whole-body planar anterior view; (B) Whole-body planar posterior view; (C) Thoracic-focused anterior view showing Perugini grade 2 myocardial uptake; (D) Thoracic-focused posterior view. In the absence of monoclonal gammopathy, this pattern is highly suggestive of transthyretin cardiac amyloidosis. 99mTc-DPD: Technetium-99m-labelled 3,3-diphosphono-1,2-propanodicarboxylic acid.

Bone scintigraphy with technetium-labeled tracer (99mTc-DPD) demonstrated Perugini grade 2 [9] myocardial uptake (Figure 5), consistent with ATTR-CM in the absence of monoclonal gammopathy.

Following multidisciplinary discussion and confirmation of ATTR-CM by non-invasive criteria, tafamidis therapy was initiated. Given the patient’s age at presentation, genetic testing was initiated to assess for hATTR; however, the results were not available at the time of manuscript submission. 

## Discussion

Cardiac amyloidosis remains a diagnostic challenge and is often missed, especially when patients present with reduced systolic function. ATTR is classically associated with older adults, with wtATTR most commonly reported in men older than 65 years [1]. When typical cardiac features occur at a younger age, hATTR becomes an important diagnostic consideration.

In our patient, echocardiography demonstrated a constellation of findings compatible with infiltrative cardiomyopathy, including concentric wall thickening, marked biatrial enlargement, restrictive filling, and a small pericardial effusion [2]. Such features can be misattributed to hypertensive heart disease or hypertrophic cardiomyopathy if the broader clinical context is not considered. The absence of longstanding hypertension or significant valvular disease strengthened the suspicion of amyloid cardiomyopathy.

Bone scintigraphy with technetium-labeled tracers has become central to the non-invasive assessment of ATTR-CM. In the absence of monoclonal gammopathy, Perugini grade 2 myocardial uptake is widely considered highly supportive of ATTR-CM and may preclude the need for endomyocardial biopsy in many patients [6,10]. Accordingly, endomyocardial biopsy was not performed in our case, as non-invasive criteria supported the diagnosis of ATTR-CM (Perugini grade 2 tracer uptake with no evidence of monoclonal gammopathy). Cardiac magnetic resonance imaging was considered to further characterize myocardial tissue involvement and provide additional prognostic information. However, it was not performed due to limited availability and the patient’s clinical status [5].

Although early cardiac amyloidosis commonly presents with preserved LVEF, systolic impairment may develop as myocardial infiltration progresses [11]. Progressive extracellular deposition can reduce myocardial contractile reserve and contribute to systolic dysfunction in advanced disease [11]. The LVEF of 35% observed in our patient suggests substantial cardiac involvement and is generally associated with a less favorable prognosis [12]. In ATTR-CM, reduced LVEF generally reflects advanced myocardial involvement and is associated with poorer prognosis compared with typical HFpEF presentations [12]. Alternative contributors to reduced LVEF (e.g., tachycardia-mediated cardiomyopathy or ischemia) should also be considered. In our patient, no sustained tachyarrhythmia was documented on the baseline assessment, and echocardiography did not show regional wall-motion abnormalities suggestive of ischemia.

The patient’s age at presentation and the severity of cardiac involvement raise suspicion for a hereditary TTR variant. Genetic testing is therefore important for diagnostic clarification and for enabling cascade screening of relatives [13]. Genetic testing was initiated; however, results were not available at the time of manuscript submission, which represents a limitation of this report and precludes definitive differentiation between hereditary and wild-type ATTR. Given the patient's young age, extracardiac features suggestive of hATTR (including neuropathy, autonomic symptoms, and carpal tunnel syndrome) were actively screened on history and physical examination. Among the numerous known TTR variants, some mutations (e.g., Val122Ile and Thr60Ala) have been frequently linked to predominantly cardiac phenotypes [14].

Timely identification of ATTR-CM has direct therapeutic implications, particularly in younger patients, as early recognition enables prompt initiation of disease-modifying therapy, supports referral for genetic counseling, and facilitates family screening when hereditary disease is suspected [2,13]. Tafamidis has been shown to reduce mortality and cardiovascular hospitalizations in ATTR-CM by stabilizing transthyretin [7]. In selected patients, particularly those with concomitant polyneuropathy or mixed phenotypes, TTR gene-silencing therapies such as patisiran and inotersen may also be considered [15,16]. In our case, therapeutic anticoagulation was initiated for right atrial thrombosis; this decision was further supported by the increased thromboembolic risk associated with cardiac amyloidosis due to atrial enlargement and mechanical dysfunction.

At the time of manuscript submission, follow-up after tafamidis initiation was not yet available, as the patient had only recently started therapy; ongoing outpatient follow-up is planned to assess clinical response and clinical trajectory. This case underscores that ATTR-CM should not be regarded exclusively as a disease of the elderly and may present at a younger age with advanced systolic dysfunction. Overall, this case reinforces the need to include ATTR-CM in the differential diagnosis of younger patients with unexplained heart failure and increased wall thickness, while keeping hATTR in mind and pursuing genetic evaluation when feasible [2,13].

## Conclusions

ATTR-CM should be considered in patients presenting with unexplained heart failure, increased ventricular wall thickness in the absence of longstanding hypertension, and discordant electrocardiographic findings such as low QRS voltages. Importantly, this diagnosis should not be dismissed solely on the basis of age, as ATTR-CM can occur in relatively younger patients and may present with atypical phenotypes, including reduced LVEF. Early clinical recognition is crucial because timely diagnosis can alter management and improve outcomes in an era of disease-modifying therapies.

Non-invasive diagnostic strategies have become central to the evaluation of suspected ATTR-CM. In particular, technetium-labeled bone scintigraphy demonstrating significant myocardial uptake, in the absence of monoclonal gammopathy, enables a reliable diagnosis without the need for endomyocardial biopsy in many cases and supports early initiation of targeted therapy. When hATTR is suspected, genetic testing remains essential to confirm the diagnosis, clarify the subtype, and facilitate appropriate family counseling and cascade screening.
